# Novel Aβ peptide immunogens modulate plaque pathology and inflammation in a murine model of Alzheimer's disease

**DOI:** 10.1186/1742-2094-2-28

**Published:** 2005-12-07

**Authors:** Jun Zhou, Maria I Fonseca, Rakez Kayed, Irma Hernandez, Scott D Webster, Ozkan Yazan, David H Cribbs, Charles G Glabe, Andrea J Tenner

**Affiliations:** 1 Department of Molecular Biology and Biochemistry, University of California, Irvine, CA 92697, USA; 2 Clarient, Inc., San Juan Capistrano, CA 92675, USA; 3 Department of Neurology, University of California, Irvine, College of Medicine, Irvine, CA 92697, USA; 4 Institute for Brain Aging and Dementia, University of California, Irvine, CA 92697, USA; 5 Center for Immunology, University of California, Irvine, CA 92697, USA

## Abstract

**Background:**

Alzheimer's disease, a common dementia of the elder, is characterized by accumulation of protein amyloid deposits in the brain. Immunization to prevent this accumulation has been proposed as a therapeutic possibility, although adverse inflammatory reactions in human trials indicate the need for novel vaccination strategies.

**Method:**

Here vaccination with novel amyloid peptide immunogens was assessed in a transgenic mouse model displaying age-related accumulation of fibrillar plaques.

**Results:**

Immunization with any conformation of the amyloid peptide initiated at 12 months of age (at which time fibrillar amyloid has just begun to accumulate) showed significant decrease in total and fibrillar amyloid deposits and in glial reactivity relative to control transgenic animals. In contrast, there was no significant decrease in amyloid deposition or glial activation in mice in which vaccination was initiated at 16 months of age, despite the presence of similar levels anti-Aβ antibodies in young and old animals vaccinated with a given immunogen. Interestingly, immunization with an oligomeric conformation of Aβ was equally as effective as other amyloid peptides at reducing plaque accumulation. However, the antibodies generated by immunization with the oligomeric conformation of Aβ have more limited epitope reactivity than those generated by fAβ, and the microglial response was significantly less robust.

**Conclusion:**

These results suggest that a more specific immunogen such as oligomeric Aβ can be designed that achieves the goal of depleting amyloid while reducing potential detrimental inflammatory reactions. In addition, the data show that active immunization of older Tg2576 mice with any amyloid conformation is not as efficient at reducing amyloid accumulation and related pathology as immunization of younger mice, and that serum anti-amyloid antibody levels are not quantitatively related to reduced amyloid-associated pathology.

## Background

Alzheimer's disease (AD) is an age-related common dementia or loss of cognitive abilities. Neuronal loss, neurofibrillar tangles and senile plaques, abnormal protein deposits which include cleavage products of the amyloid precursor protein (amyloid β peptides (Aβ)) are pathologic characteristics of the disease. While the mechanism of this neurodegeneration remains to be defined, substantial evidence implicating a significant role for the Aβ peptide (40–42 amino acids) has been reported (reviewed in [1,2]). As a result, one general therapeutic approach being investigated is the reduction of amyloid peptide accumulation in the brain. Several reports have shown that when mice containing the transgene for human mutant amyloid precursor protein (APP) were immunized with fibrillar Aβ peptide prior to the accumulation of amyloid deposits, Aβ deposition observed at later ages was greatly decreased [3-6]. However, when applied to humans, "immunization" with Aβ resulted in the development of an adverse inflammatory reaction in a fraction of the patients [7-9], which led to a reevaluation of this strategy for AD in humans, particularly at that stage of the disease when substantial fibrillar amyloid deposits have begun to accumulate [10]. It is this stage of the disease that often correlates with appearance of cognitive deficiencies that is a defined point at which potential therapy may be initiated.

Several studies in mouse models have shown that passive immunization, in these cases intracranial or peripheral injection of anti-Aβ antibodies, resulted in relatively rapid clearance of significant amounts of Aβ immunoreactivity, both extracellular deposits as well as intraneuronal Aβ accumulation [11-15]. Furthermore, decreases in amyloid accumulation by either passive or active immunization are accompanied by improvement of cognitive function in these murine models [16,17] and previous work reviewed in [18]). However, not all anti-amyloid antibodies provide the same degree of protection [19], and there have been at least two reports in which animals with established robust plaque load did not respond to a particular immunogen [3,20]. Thus, as with other immunological responses, the nature of the immunogen, the adjuvant used for immunization, the age and the genetics of the animals immunized all contribute to defining the immune response that subsequently develops and these differences lead to various degrees of clearance and protection from injury.

Recent reports have defined an oligomeric conformation of the Aβ structure that alters LTP activity [21,22] and induces neurotoxicity *in vitro *that can be reversed by addition of anti-oligomeric antibody [23,24]. Since Aβ oligomers are proposed to be an intermediary conformation prior to fibril formation and it has been proposed that antibodies preventing or reversing amyloid assemblies may be therapeutic [25-27], we tested immunization with a novel immunogen presenting the oligomeric conformation of Aβ [23]. In addition, the Aβ oligomers may be more transient (and present at lower concentrations at any given time) than other conformations, and thus immunization with the oligomeric form of the amyloid peptide may provide benefit with minimal induction of inflammatory cascade. The data obtained demonstrate that immunization with an oligomeric conformation of the peptide is as efficient as immunization with either fibrillar amyloid or a multiple antigen peptide amyloid immunogen in terms of clearing amyloid, and that microglial reactivity is significantly less with oligomers as the immunogen than other amyloid conformations.

The experiments described here were also designed to assess the effect of immunization of animals at an advanced age/stage of pathology on the mitigation of amyloid associated neuropathology in a mouse overexpressing human mutant amyloid precursor protein, and to determine whether differences in complement deposition could be detected on plaques resistant to clearance. Our results, in addition to identifying a novel candidate immunogen, demonstrate that while the level of measured serum antibodies are similar or only slightly different in animals immunized with a given immunogen at different ages, a decrease in the accumulation of both fibrillar and diffuse amyloid plaques occurs only when mice are immunized at early stages of the disease (12–16 months of age). The level of C3 activation fragments associated with plaques was also reduced in animals immunized with any amyloid immunogen, correlating with reduced fibrillar plaque burden. Finally, a new, automated, computer assisted method of quantification of immunoreactivity is described and shown to correlate well with conventional image analysis.

## Methods

### Amyloid peptide fibril and oligomer preparation

Lyophilized Aβ1–42 peptides were resuspended in 50% acetonitrile in water and re-lyophilized. Soluble oligomers were prepared by dissolving 1.0 mg of peptide in 400 μL hexafluoroisopropanol (HFIP) for 10–20 min at room temperature. 100 μl of the resulting seedless solution was added to 900 μl MilliQ H_2_O in a siliconized Eppendorf tube. After 10–20 min incubation at room temperature, the samples were centrifuged for 15 min. at 14,000 × G and the supernatant fraction (pH 2.8–3.5) was transferred to a new siliconized tube and subjected to a gentle stream of N_2 _for 5–10 min to evaporate the HFIP. The samples were then stirred at 500 RPM using a Teflon coated micro stir bar for 24–48 hr at 22°C. Oligomers were validated by atomic force microscopy (AFM), electron microscopy (EM) and size exclusion chromatography (SEC) as described [23]. Fibrils are formed by stirring the same solution for 7 days. Fibrils were sedimented and washed in PBS, and resuspended at 2 mg/ml. Fibrillar β amyloid (fAβ) peptides were stored at -70°C until immunization. For the oligomer antigen (oligo), Aβ oligomer molecular mimic was prepared by conjugating Aβ40 via a carboxyl terminal thioester to 5 nm colloidal gold as previously described [23], and stored at 4°C until used. A multiple antigen peptide (MAP) which contains a core matrix of 4 branching lysines contiguous with the amyloid beta 1–33 peptide (ie.MAPAβ_1–33_) containing both the native B and T cell epitopes of Aβ was synthesized (Invitrogen Inc., Carlsbad, CA) to increase the response to Aβ. Peptides were resuspended in sterile PBS at 2 mg/ml, vortexed and stored at -70°C.

### Animals and immunization scheme

Tg (HuAPP695.K670N-M671L)2576 mice from K. Hsiao [28] and non-transgenic littermates or B6/SJL wild type mice were used as controls. fAβ or oligomer mimic were emulsified 1:1 (v/v) with complete Freund's adjuvant (CFA) for the first immunization, while MAPAβ_1–33 _peptides were emulsified 1:1 (v/v) with complete Freund's adjuvant containing 4 mg/ml Mycobacterium tuberculosis (Difco, Voight Global, Kansas City, Mo) [29]. Subsequent immunizations with each immunogen in incomplete Freund's adjuvant (IFA) were performed after 2 weeks, and monthly thereafter for 3 additional injections. Two weeks after the final immunization, animals were bled and perfused as described below. In all immunizations 100 ug peptide was injected subcutaneously per mouse. In addition, at the time of initial immunization with MAPAβ_1–33 _500 ng of pertussis toxin (PTX) (Sigma, St. Louis, MO) in 200 ul PBS was injected IP, followed by a second injection 24 hours later [29]. Immunization controls for both wild type and transgenic mice included injections of adjuvant with PBS only (no peptide antigen). All experimental procedures were carried out under protocols approved by the University of California Irvine Institutional Animal Care and Use Committee.

### Tissue collection and immunohistochemistry

Mice were deeply anesthetized with an overdose of pentobarbital (150 mg/kg, IP), blood collected by cardiac puncture, and then animals perfused transcardially with cold phosphate-buffered saline (PBS). After dissection, brain tissue was fixed overnight with 4% paraformaldehyde in PBS, pH 7.4 at 4°C. Thereafter, fixed tissue was stored in PBS/0.02% sodium azide (NaN_3_) at 4°C until use. Fixed brain tissue was sectioned (40 um) with a vibratome, and coronal sections were collected in PBS (containing 0.02% sodium azide), and stored at 4°C prior to staining.

Immunohistochemistry (IHC) was performed on free-floating brain sections. To stain for Aβ plaques, sections were immersed in 50% formic acid for 5 min. Endogenous peroxidase in tissue was blocked by treating with 3% H_2_O_2 _in PBS, 10 min at room temperature. Nonspecific background staining was blocked by1 hour incubation in 2% BSA with 0.3% Triton X-100 (TX) at room temperature. Sections were then incubated with primary antibodies (Table 1) overnight at 4°C, rinsed 3 times with PBS with 0.1% TX and incubated with biotinylated secondary antibody followed by ABC kit reagent (Vector, Burlingame, CA) for 1 hour each at room temperature. Finally, after washing three times, the sections were incubated for approximately 2~5 min with diamino-benzidine (DAB), (Vector). Sections were mounted on slides, dehydrated in a series of graded ethanol, cleared with xylene, and then coverslipped with DePeX (Biomedical Specialities, CA). For fluorescent staining, biotinylated secondary was detected by incubation with Streptavidin-CY3 (Vector) for 1 h at RT. Fibrillar Aβ was visualized by incubating the sections in 1% thioflavine for 30 minutes followed by a 1 minute wash in 50% ethanol, 5 min in deionized distilled water, and 5 min in PBS. Sections incubated in parallel without primary antibody or IgG control did not develop staining.

**Table 1:** Summary of antibodies used in this study

Antibody	Antigen	Type	Source	Dilution^A^	Reference
GFAP	Glial Fibrillary acidic protein (bovine)	Rabbit polyclonal	Dako	IHC: 4 ug/ml	[58]
6E10	Aβ1–17 (human)	Mouse monoclonal	Seneteck	IHC: 1 ug/ml	[59]
MAC-1	CD11b (mouse)	Rat monoclonal	Serotec	IHC: 10 ug/ml	[60]
CD45	CD45 (mouse)	Rat Monoclonal	Serotec	IHC: 1 ug/ml	[61]
C3(2/16)	C3/iC3b/C3c (mouse)	Rat Monoclonal	Lambris	IHC 1:500	[36]
C3(2/11)	C3b/iC3b/C3c (mouse)	Rat Monoclonal	Lambris	IHC 1:1000	[36]
M-16	β-Amyloid	Rabbit Polyclonal	Glabe	IHC 1:2000	[62]

^A ^IHC, immunohistochemistry

### Image analysis

Immunostaining was observed under a Zeiss Axiovert-200 inverted microscope (Carl Zeiss, Thornwood NY) and images acquired with a Zeiss Axiocam high-resolution digital color camera (1300 × 1030 pixel) using Axiovision 3.1 software. Digital images were analyzed using KS300 analysis program (Zeiss). Percentage of immunostained area (area of immunostaining/total image area × 100) was determined for all the markers studied by averaging % Field Area of several images per section that cover most, or all, of the region of study. Assays were repeated at least twice, with n = 4–7 animals per group per age per marker as noted in legends and text. Quantitative comparisons were performed on sections processed at the same time. Single ANOVA statistical analysis was used to assess the significance of the differences in plaque area, glial and C3 activation products reactivity among the animals groups.

A second method of quantification developed for the ACIS image analysis system (Clarient, Inc., San Juan Capistrano, CA) was utilized to analyze the 6E10 immunoreactivity. Images were acquired automatically. Cortical and hippocampal regions appropriate for analysis were selected and automatically scored using an algorithm that identifies objects based on user-configurable parameters. Object identification was paired with a watershed segmentation algorithm to facilitate separation of touching and overlapping deposits. In this manner large deposits that form contiguous bands of Aβ were separated into individual objects. Because Aβ deposits in these animals can vary markedly in size and shape, the identification of Aβ-positive objects utilized a broad size filter (12–3,000 microns effective diameter) and did not employ rigorous morphometric filters. Data collected for each Region of Interest (ROI) were the area of tissue scored (area of the ROI), the number of Aβ-positive objects identified and the total area of the Aβ-positive objects. These parameters allowed calculation of two different measures of amyloid load: the Aβ-Positive Object Density, which is simply the number of objects per mm^2 ^of tissue scored and is used as an approximation of the number of plaque-like structures per mm^2^, and secondly, the ratio (as a percent) of the total area of the Aβ-positive objects to the area of tissue scored. It should be noted that, since the numerator of this second ratio contains only the area enclosed within the boundaries of the identified objects and does not incorporate small particles of Aβ-immunoreactivity that are excluded by the size filter (i.e. <12 microns effective diameter), this measure is distinct from the area ratio described in the previous section and will thus be denoted as the Aβ-Positive Object Area Ratio.

Comparisons among experimental groups were based on single values per animal for Aβ-Positive Object Density and Aβ-Positive Object Area Ratio. These were calculated by determining the sum of the numbers of Aβ-positive objects (or the sum of the areas of the Aβ positive objects) for the entire section and dividing by the sum of the areas of all ROIs. This analysis was performed blinded to results from prior conventional quantification as described above.

### ELISA analysis of anti Aβ antibodies

ELISA assays were performed as previously described [23]. Briefly, 50 ng/100 ul of monomeric, oligomeric or fibrillar Aβ40 was plated on ELISA wells and blocked with BSA. Serum samples were initially diluted 250-fold and then serially diluted two-fold to an end point of 1:64,000. The secondary antibodies used for detection were peroxidase-conjugated AffiniPure Goat Anti-mouse IgG (H+L) (Jackson ImmunoResearch) and peroxidase-conjugated anti-mouse IgM (Zymed/Invitrogen, Carlsbad, CA). For samples where the absorbance exceeded 3 times the background absorbance, the titer was determined from the midpoint of the dilution curve (IC50). For samples that did not exceed this criterion, the titer was assumed to be less than the initial dilution of 1:250.

## Results

### Immunization initiated at 12 months of age with any Aβ conformation decreased both total and fibrillar Aβ immunostaining in Tg2576 mice

**Figure 1:**
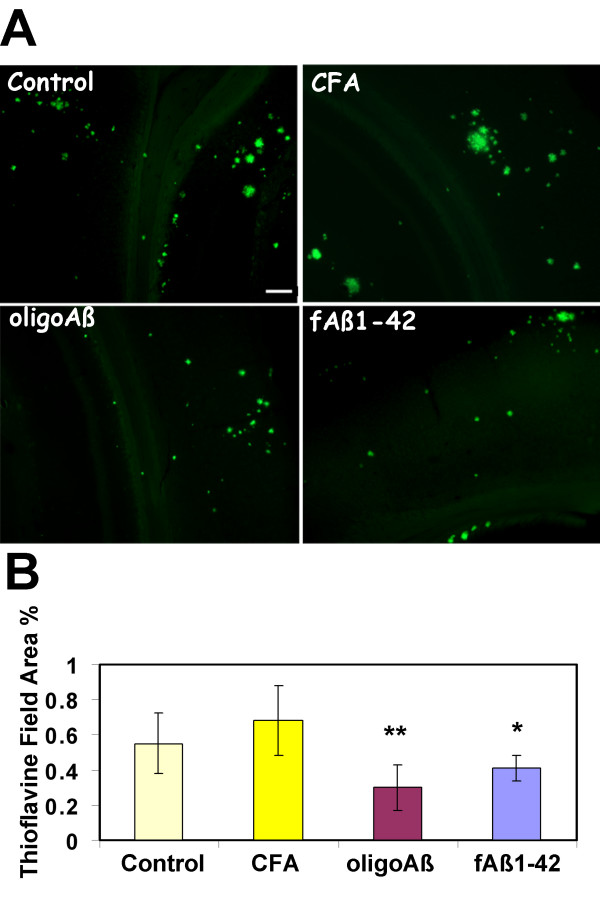
**Thioflavine positive plaques are decreased in TG 2576 mice immunized from 12–16 months with oligomeric or fibrillar Aβ conformations**. A. Cortex of Tg2576 at 16 months was stained with thioflavine as described in Materials and Methods (control: untreated, CFA: adjuvant only, Oligo Aβ: colloidal gold conjugated amyloid β, and fAβ1–42: fibrillar amyloid). Scale bar = 100 microns. B. Image analysis of thioflavine in hippocampus and cortex of animals immunized at 12–16 months. Mean of each animal is the average of 2–4 sections (except untreated control which is 1 section per animal) in which most to all of the area of study was analyzed (total 4–8 images per section). Bars represent group mean ± SD of n mice per group: Control n = 9, CFA n = 4, oligo Aβ n = 7, Aβ n = 5. *p < 0.02, **p < 0.005 by ANOVA relative to CFA (adjuvant only) control.

**Figure 2:**
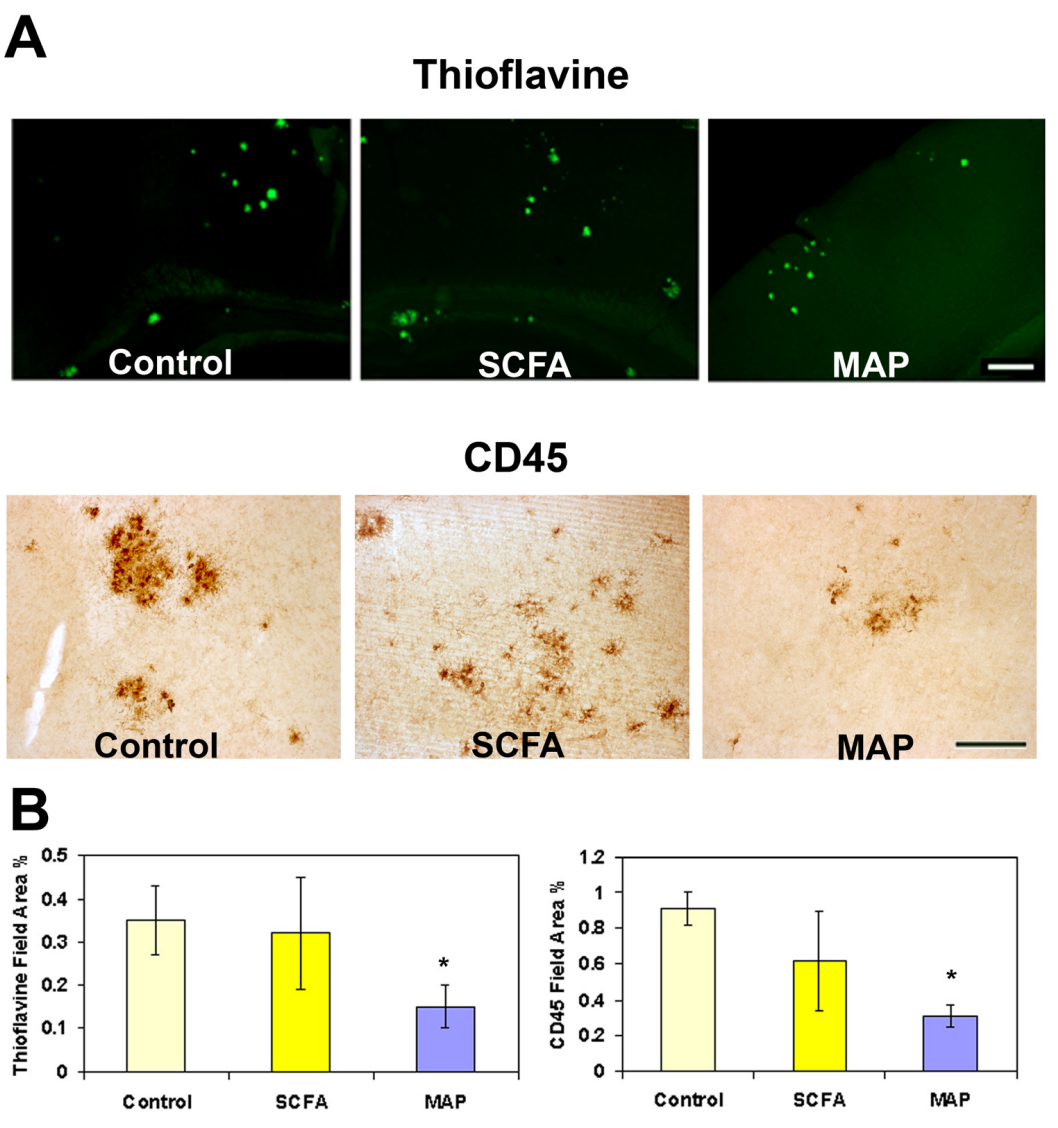
**Immunization of animals at 12–16 months with MAPAβ1–33 decreases thioflavine and anti CD45 reactivity**. A. Representative photomicrographs of thioflavine (green) or CD45 (brown) staining of brain sections from 16 months Tg2576 untreated (control), or injected with adjuvant alone (SCFA) or MAPAβ_1–33 _(MAP). Scale bar 100 microns (upper panel) and 50 microns (lower panel). B. Image analysis of thioflavine staining or CD45 immunoreactivity in cortex and hippocampus of animals untreated or immunized at 12–16 months. Mean of each animal is the average of two sections (stained in two independent assays, each of which contained animals from each treatment group) in which most to all the area of study was analyzed (4–8 images per section). Bars represent mean +/- SD of n mice per group. Thioflavine: Control n = 6, SCFA n = 6, MAP n = 5, *p < 0.02; CD45: Control n = 5, SCFA n = 7, MAP n = 5, * p < 0.04.

In AD significant cognitive decline is generally correlated with the appearance of "mature" amyloid plaques [26,30]. These plaques contain fibrillar amyloid peptide and as such can be detected by thioflavine, a reagent that stains proteins in beta sheet conformation [31]. Therefore, the accumulation of thioflavine stained plaques in animals immunized at ages 12–16 months was assessed in hippocampus and cortical regions of control animals or animals immunized from 12–16 months of age with fAβ, oligo Aβ, MAPAβ_1–33 _or adjuvant alone. Representative photomicrographs presented in Figure 1A, and image analysis using KS300 analysis program (Zeiss) of sections from multiple animals demonstrated that the mean % thioflavine positive area in oligo Aβ and fAβ immunized groups (sacrificed at 16 months of age) was decreased by 56 and 40% respectively, relative to adjuvant control. Similarly, when MAPAβ_1–33 _was used as an immunogen, fibrillar plaque accumulation was decreased by 53% relative to adjuvant control (Figure 2). There was no significant difference between untreated controls and animals treated with adjuvant only (Figure 1A and 1B) or adjuvant plus pertussis toxin in thioflavine positive plaques (Figure 2A and 2B) or any of the markers tested below.

**Figure 3:**
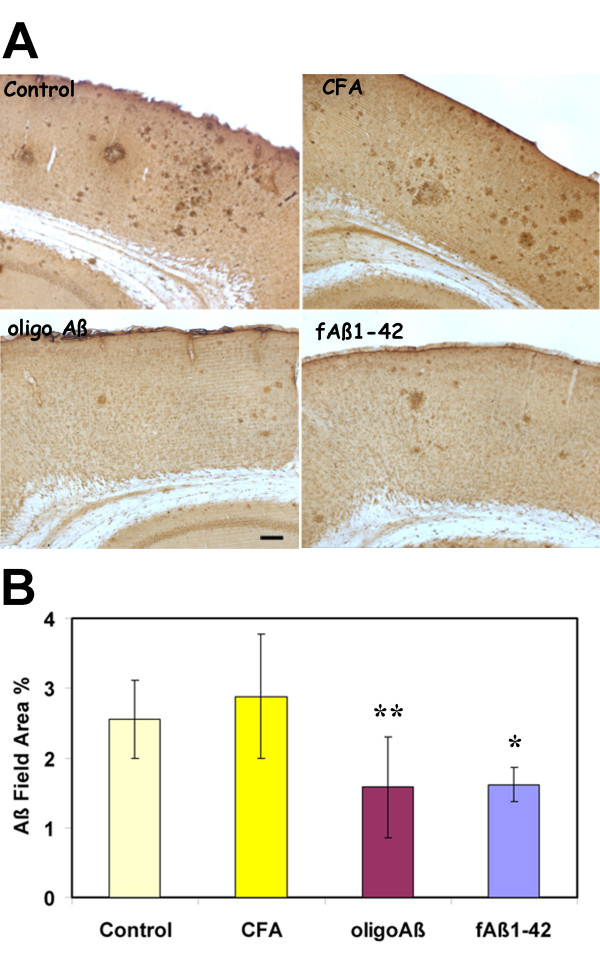
**Immunization initiated at 12 months of age in Tg2576 animals decreases total amyloid deposits**. A. Representative photomicrographs of sections from brains of 16 m Tg2576 (Control, CFA, Oligo Aβ and fAβ) that had been immunized as described in Materials & Methods from 12 to 16 months of age immunostained with 6E10 (which reacts with the human amyloid peptide). Scale bar: 100 microns. B. Image analysis (% Field area) of Aβ (6E10 antibody) immunoreactivity in hippocampus and cortex of animals untreated or immunized at 12–16 months. Mean of each animal is the average of 2 sections (except untreated control which is 1 section per animal) assessing 4–8 images per section (most to all of the area of the section was analyzed). Bars represent group mean ± SD of n mice per group: Control n = 6, CFA n = 4, Oligo Aβ n = 7, Aβ n = 4, *p < 0.04, **p < 0.03 by ANOVA.

To assess total amyloid deposits in the APP transgenic mice after the 4 month regime of immunization, the human Aβ specific monoclonal antibody, 6E10, was used as described in Material and Methods. Figure 3 shows representative photomicrographs from the cortex and hippocampal region of control or immunized animals. Image analysis of sections from multiple animals demonstrated that the mean % field stained area (ie. Aβ deposits) in oligo Aβ and fAβ immunized groups was decreased by greater than 44% relative to the CFA/IFA controls (Figure 3). Immunization with MAPAβ_1–33 _decreased total amyloid deposition (6E10 staining) by 52% and 44% relative to adjuvant and untreated control respectively (data not shown, n = 6 animals per group, p < 0.03).

To further validate the quantification of these immunohistochemical results, sections were also analyzed using an automated digital imaging system (ACIS, CLARiENT). A digital image was acquired for the entirety of each section at a resolution of one pixel per micron. All cortical and hippocampal tissues were then analyzed by object identification and feature extraction algorithms. Since analysis of over 50 sections containing both cortex and hippocampus demonstrated that immunoreactivity was higher in the cortex than in the hippocampus, only sections containing cortex and hippocampus were used in these treatment comparisons. Both oligomeric and fibrillar Aβ conformations, as well as the MAPAβ_1–33 _immunogen, resulted in significant changes in Aβ deposition, with 2- and 3-fold reductions in both plaque number (Aβ-Positive Object Density) and plaque area (Aβ-Positive Object Area Ratio) relative to the respective adjuvant controls (*p *< 0.05 by two-tailed *t*-test). In particular, the extent of the reduction in the novel measure of Object Density by Aβ, oligo or the MAPAβ_1–33 _immunized groups was essentially identical compared to the adjuvant controls, (ie. reductions in plaque number were 2.73, 2.76 and 2.63-fold respectively, p < 0.01, one tail t-test). The significant decreases in plaque area in Aβ, oligo or the MAPAβ_1–33 _immunized groups obtained by this method were 1.96, 2.39, and 2.72-fold. These quantitative results correlated well with conventional image analysis of the same specimens (r = 0.84, *p *< 0.0001 for Aβ Positive Object Density vs. conventional Area Ratio, and r = 0.75, *p *< 0.0001 for Aβ Positive Object Area Ratio vs. conventional Area Ratio), providing additional validation to the conclusion that immunization with three different forms of the amyloid peptide result in a similar decrease in amyloid plaque accumulation in this murine model when immunized during a period of rapid amyloid deposition.

### GFAP reactivity is decreased in Tg2576 immunized at 12–16 months with oligo Aβ or fAβ

**Figure 4:**
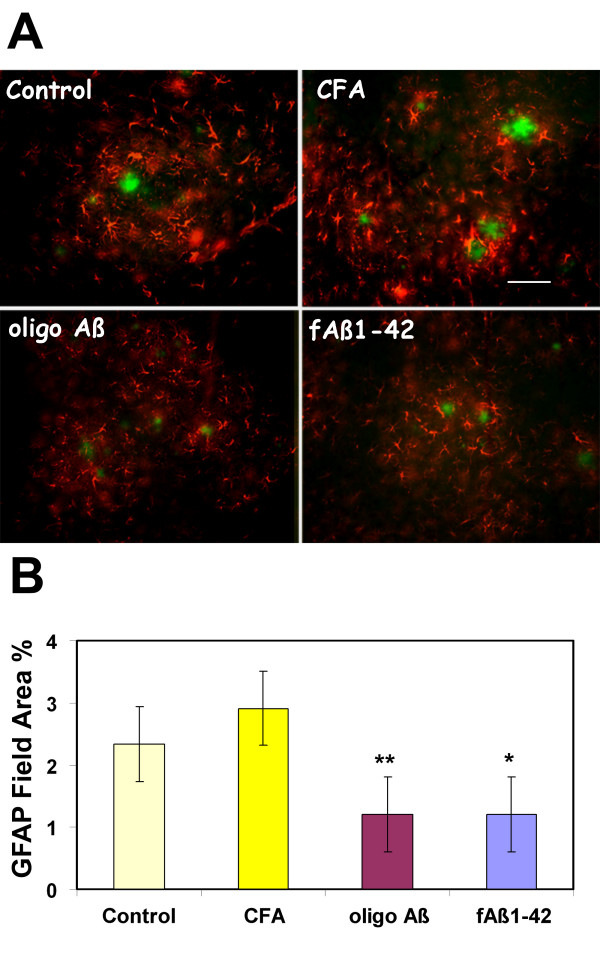
**Immunization with oligomeric or fibrillar Aβ at 12–16 months decreases astrocyte activation relative to untreated or adjuvant treated age matched controls**. A. GFAP reactivity (red) around fibrillar plaques (thioflavine, green) at 16 months in Tg2576 mice either untreated (Control), or immunized at 12–16 months of age with adjuvant only (CFA), oligo Aβ or fAβ. Scale bar = 50 microns. B. Image analysis of GFAP immunoreactivity in cortex of animals immunized at 12–16 months. Mean of each animal is the average of 2–4 sections (total 4–8 images per section) in which most to all of the area of study was analyzed. Bars represent group mean ± SD of n mice per group: untreated = 4, CFA n = 4, oligo Aβ n = 7, fAβ n = 5. *p < 0.03, **p < 0.01 by ANOVA.

Characteristic of fibrillar amyloid plaques in both human AD brain and in mouse models of amyloid accumulation is robust association of activated astrocytes, which can be envisioned by the astrocyte cell marker GFAP. GFAP staining of B6/SJL control animals was negligible relative to the APP transgenic animals, whether untreated, immunized with adjuvant only or any Aβ immunogen (data not shown). However, while prominent plaque associated GFAP staining was seen in both untreated and adjuvant only transgenic mouse brain, the mean GFAP % field area after the 4 month immunization scheme described in M & M with oligo Aβ or fAβ was 42 and 41% respectively of CFA control, ie a decrease of 58% and 59% (Figure 4A and 4B). This is consistent with the quantitative correlation between mature, fibrillar amyloid plaque accumulation and astrogliosis. That is, the amount of fibrillar plaque deposition correlates with astrogliosis.

### Immunization with oligomeric Aβ at 12–16 months resulted in less microglial immunoreactivity relative to immunization with fAβ

**Figure 5:**
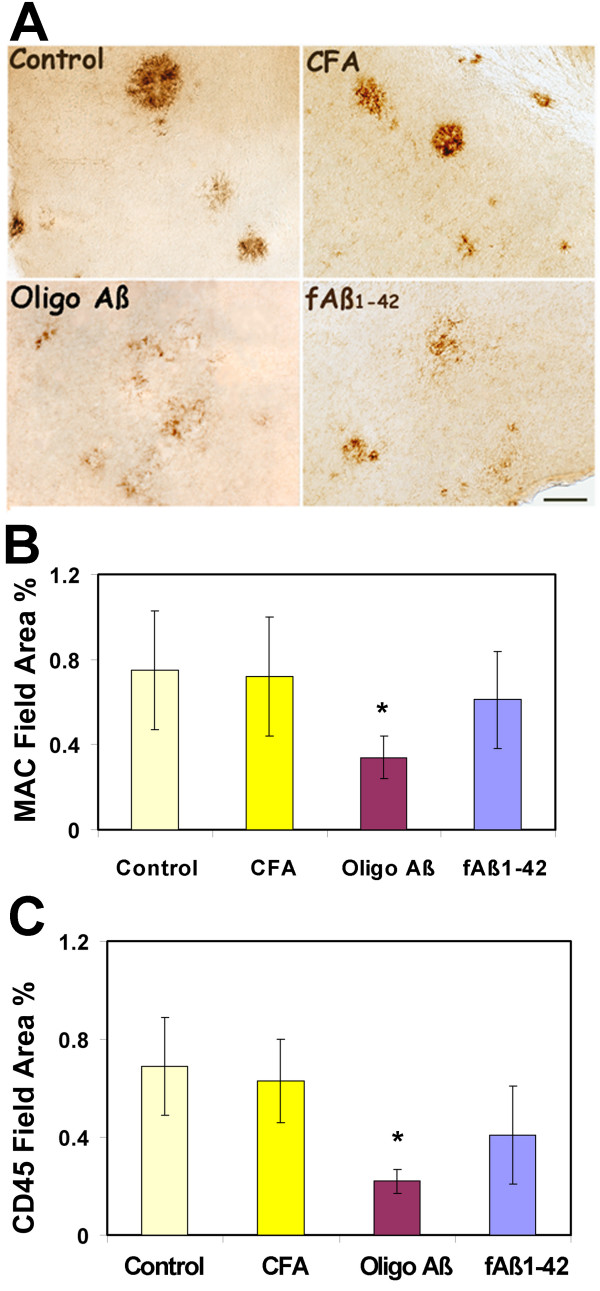
**Immunization with oligomeric Aβ at 12–16 months decreases microglia reactivity to a greater extent than immunization with fAβ**. Photomicrographs of immunohistochemical staining of microglial reactivity (MAC-1, brown) in Tg2576 untreated or immunized with CFA, Oligo Aβ and fAβ at 12–16 mos (A). Scale bar = 50 microns Image analysis of MAC-1 (B) and CD45 (C) immunoreactivity in hippocampus and cortex of animals immunized at 12–16 months. Values for each animal were the average of 6–8 images per section which resulted in analysis of most to all of the area of the section. Bars represent group mean ± SD of n mice per group: Control n = 7, CFA n = 4, oligo Aβ n = 6–7, fAβ n = 5. By ANOVA, for MAC, CFA: oligo Aβ *p < 0.01; for CD45, CFA: oligo Aβ *p < 0.001. Data for each marker are from one assay representative of 2 independent assays.

Microglia play a major role in regulating homeostasis in the brain and have the ability to actively phagocytose, to secrete cytokines, and to present antigen to T cells depending on their stimulatory environment [32]. However, some of these same neuroprotective functions can become detrimental if dysregulated [33]. Given the common observation that reactive microglia are characteristically associated with fibrillar amyloid containing plaques in both human AD and animal models, we assessed the effect of immunization on microglial reactivity as measured by CD45 and MAC-1 surface antigens. The photomicrographs from cortical brain areas show a pronounced decrease in MAC-1 in animals immunized with oligo Aβ or fAβ as compared to untreated or adjuvant treated transgenic mice (Figure 5A). Mean % area of MAC-1 staining in oligo Aβ and fAβ immunized groups is 40 and 76% respectively of CFA control (Figure 5B). Results were similar when immunoreactive CD45 was assessed with mean field area % staining in oligo Aβ and fAβ groups at 35 and 60% respectively of the CFA control (Figure 5C and data not shown). Adjuvant only (CFA) animals and untreated animals were indistinguishable. Interestingly, while there is a clear trend to lower microglial activation in the fAβ immunized animals, a statistically significant difference was not established in the group studied relative to the CFA control, perhaps due in part to the variability in the animals. However, the difference in microglial reactivity in those animals immunized with the oligomeric conformation of Aβ was statistically significant relative to either the untreated controls or adjuvant controls (p < 0.01, MAC-1; p < 0.001, CD45). Indeed, CD45 and MAC-1 reactivity in the Oligo Aβ immunized animals is significantly lower than that in the fAβ immunized animals (p < 0.02 and p < 0.05, for MAC-1 and CD45 respectively). These results could reflect a lower variability in the activation of microglia in response to immunization with oligo Aβ than with fAβ and could be related to the mechanisms resulting in the decreased accumulation of amyloid in the immunized animals in each case. Interestingly, immunization with MAPAβ_1–33 _also showed significantly less CD45 (50% of adjuvant control, p < 0.036) (Figure 2) and MAC-1 (38% of adjuvant control, p < 0.012, 1 experiment, 4–7 animals per group, data not shown).

### Immunization with any amyloid immunogen at 16–20 months shows no effect on plaque pathology

**Figure 6:**
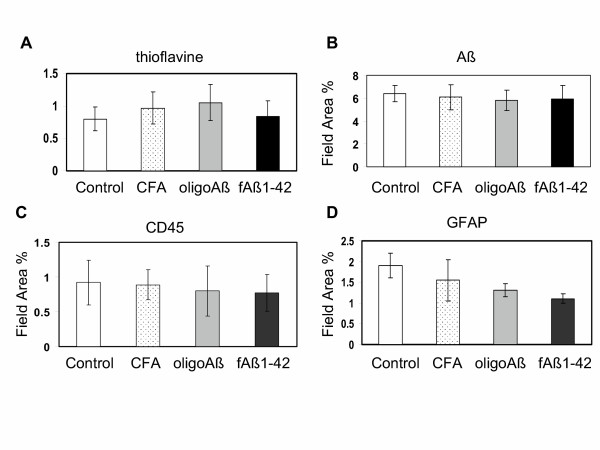
**Immunization of Tg2576 mice at 16 – 20 months provided no significant change in brain pathology**. Image analysis of immunoreactivity in sections of cortex/hippocampus of animals immunized at 16–20 months. Animals were immunized and tissue processed and stained for thioflavine (A), 6E10 (B), CD45 (C) and GFAP (D) as described above for the younger animals. Mean of each animal is the average of 2–3 assays/sections per marker, derived from 4 – 8 images per section (including most to all of the area of study). Bars represent group mean ± SD of n mice per group: Control (untreated) = 4–6, CFA n = 6, oligo Aβ n = 6–7, fAβ n = 4–6. No significant differences are seen between groups in any of the markers.

The identical immunization scheme was performed with older Tg2576 and wild type (B6/SJL) animals. Immunization was initiated at 16 months of age, a time at which there was robust total and fibrillar amyloid deposits (see untreated controls in Figures 1, 2, 3, 4, 5) in the transgenic mice, and continued through 20 months of age. Animals were then bled, sacrificed and assessed for each of the markers described above. No differences in thioflavine staining, 6E10, GFAP, MAC-1, or CD45 were seen between any treatment groups regardless of the immunogen (Figure 6 and data not shown for MAC-1 and the MAPAβ_1–33 _immunized animals).

### Immunization with oligomeric Aβ conformations induced measurable antibody reactivity to oligomers but not to soluble or fibrillar amyloid

Levels of anti-amyloid antibody induced in all immunized animals differed to some extent among the immunogen and the age of animals at the time of immunization, although all animals responded to immunization. A most notable observation was that immunization of both transgenic and wild type mice with the oligomeric conformation of Aβ resulted in measurable antibody to the oligomeric form of Aβ (Table 2), with trace to no reactivity to fibrillar Aβ or soluble Aβ as assessed by conformation specific ELISA. Immunization with fAβ or Aβ_1–33 _as a MAP peptide generated significant levels of IgG anti-oligomeric Aβ as detected by ELISA (Table 2), but also induced antibody to fAβ and to soluble Aβ (data not shown). The transgenic animals immunized with fAβ or oligo Aβ at a younger age (12–16 m) had slightly higher antibody titers than those immunized later (16–20 m), although the level of antibody in animals immunized with MAPAβ_1–33 _was not related to age of immunization. Nontransgenic immunized mice had greater responses to all immunogens relative to the transgenic mice, and those responses did not vary according to the age of immunization. However, the level of anti-oligomeric IgG antibody or any anti Aβ antibody had no detectable correlation with any of the pathologic markers at either age group when individual transgenic animals were compared (data not shown). Furthermore, as would be expected after 4 months of immunization, no appreciable IgM anti oligomeric Aβ or anti fibrillar Aβ was detected (using an ELISA specific for IgM detection). Only very low levels of IgM anti soluble Aβ was observed in sera from animals immunized with fibrillar Aβ.

**Table 2:** IC50 Anti Oligomeric Aβ

		**12–16 m**			**16–20 m**		
**TG2576**		Average	SD	n	Average	SD	n
			
	CFA	<250		3	<250		5
	Oligo	894	122	4	444	75	5
	fAβ	1463	75	6	1270	336	5
							
	SCFA	<250		5	<250		6
	MAPAβ_1–33_	807	138	5	890	200	6
							
**Wild Type**		Average	SD	n	Average	SD	n
			
	CFA	<250		6	<250		2
	Oligo	1051	246	4	1311	305	5
	fAβ	2385	350	5	2267	248	7
							
	SCFA	<250		1	<250		2
	MAPAβ_1–33_	1746	72	5	1586	178	4

To determine if the antibodies generated with each immunogen were able to bind amyloid plaques in the APP transgenic mice, dilutions of sera from each group of animals immunized at 12–16 months and animals immunized with fAβ at 16–20 months were screened for the ability to bind to amyloid deposits in brain sections from an unimmunized 16 month APP mouse. Consistent with the ELISA data above, antisera from oligo immunized animals did not stain plaques, whereas antisera from animals immunized at 12–16 months and 16–20 months with fAβ and sera from MAPAβ_1–33 _animals all stained cortical plaques (data not shown).

### Mouse IgG is detected colocalized with plaques in Tg 2576 immunized at 12 months with fAβ

**Figure 7:**
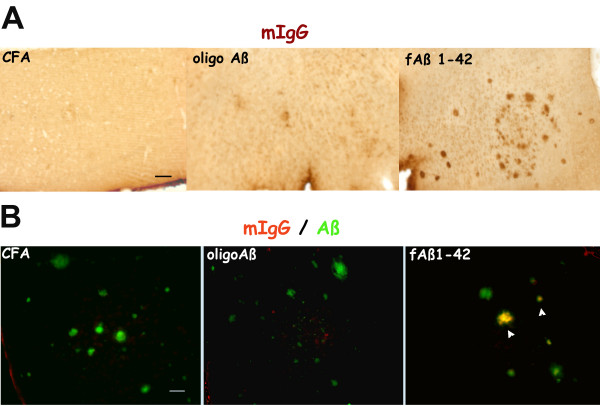
**IgG is deposited at detectable levels in Tg 2576 animals immunized at 12 months with fAβ**. Representative pictures of cortex sections of mice immunized from 12–16 months with CFA, Oligo Aβ and fAβ. A. Direct immunoperoxidase labeling of mouse IgG using a biotinylated anti mouse IgG and streptavidin-HRP. B. Fluorescent double labeling with horse biotinylated anti mouse IgG (Vector) detected with streptavidin-CY-3 (red) and polyclonal anti Aβ antibody (M-16) detected with FITC conjugated anti rabbit antibody (green). Arrowheads show colocalization of reactivity in some of the plaques. Scale bars: 50 microns.

As an indication of whether any induced antibody to β-amyloid had access to the brain, sections from brain of immunized animals were tested for immunoreactivity to mouse IgG using two approaches: direct staining with biotinylated anti mouse IgG (Figure 7A) and indirect immunohistochemistry using rabbit anti mouse IgG as a primary antibody and subsequently probing with anti rabbit immunoglobulin (data not shown). Both staining approaches gave comparable results. Deposits of IgG were detected in cortex/hippocampus sections of animals immunized with fAβ at 12 months (Figure 7A) and these deposits of mIgG colocalized with Aβ positive plaques (Figure 7B). All animals immunized with fAβ at 12–16 months that were tested (n = 6) showed these deposits. Of the animals tested immunized with the oligo Aβ (n = 7) only one had slight IgG reactivity (Figure 7A) within the cortex, while no staining was detected on any of the adjuvant controls (Figure 7A, n = 5). Interestingly, none of the animals immunized at 16 months showed any IgG deposited in the brain (data not shown, CFA controls = 3, oligo Aβ n = 5, fAβ n = 5). All the animals immunized with SCFA (n = 4) or MAPAβ_1–33_(n = 4) at 12–16 months that were tested were negative for plaque-associated mouse IgG. Sections from each animal were tested 2–4 times. Thus, only animals immunized with fAβ at 12–16 months showed any plaque-like IgG deposition in brain tissue, even though the titer of anti fibrils and anti-soluble Aβ, were generally higher in the MAPAβ_1–33 _immunized animals.

### Complement C3 protein correlates with plaque pathology

**Figure 8:**
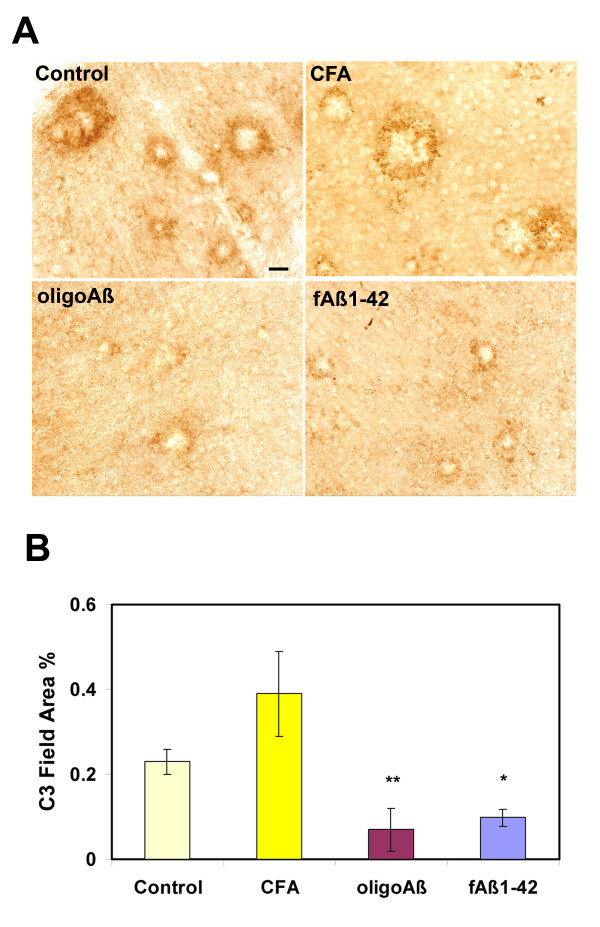
**Anti murine activated C3 is detected in plaque-like structures in Tg2576 and is decreased in animals immunized with either fAβ or oligo Aβ**. A. Photomicrographs of immunohistochemical straining with 2/16 anti mouse C3b/iC3b (brown) in Tg2576 untreated or immunized with CFA, Oligo Aβ and fAβ at 12–16 mo. Scale bar = 20 microns. B. Anti C3b/iC3b immunoreactivity in animals immunized at 12–16 months. Values for each animal were the average of 6–8 images per section which resulted in analysis of most to all of the area of the section. Bars represent group mean ± SD of n mice per group: Control n = 4, CFA n = 3, oligo Aβ n = 6, fAβ n = 5. *p < 0.002, **p < 0.001 relative to CFA, by ANOVA.

Complement activation products associated with plaques have been detected in murine models of AD [34,35]. Since antibody-antigen complexes are known to activate complement, it was hypothesized that complement activation on plaques with associated antibodies (ie. those immunized with fAβ at 12–16 months) may initiate the complement cascade thereby increasing the amount of C3 bound to the plaques (and thereby possibly contributing to greater microglial reactivity). Novel monoclonal antibodies have recently become available that specifically recognize C3 activation products (C3b, iC3b and C3c) [36]. These antibodies (2/16 and 2/11) were used to compare C3 activation products in brain sections from mice immunized with oligo Aβ and fAβ or with adjuvant alone. C3b/iC3b immunoreactivity was detected in all brain sections of Tg2576 mice (Figure 8A). Interestingly, results of image analysis demonstrated that the level of activated C3 correlated with fibrillar Aβ plaque load (Figure 8B). That is, no apparent increase in C3 deposition was detected in those animals immunized with fAβ and shown to have antibody associated with the plaques. Rather, the amount of C3 reactivity reflected the reduced quantity of plaques present in the immunized animals relative to the adjuvant control animals, and was nearly identical to that seen in similar brain sections of animals immunized with oligo Aβ. C3 staining in animals immunized at 16–20 months correlated with thioflavine staining with no increase or decrease in C3 deposition detectable in any of the animals (data not shown).

## Discussion

The data presented here is the first investigation of the effect of active immunization with amyloid beta peptide in a specific oligomeric conformation in a murine mouse model of Alzheimer's Disease. Immunization with this form of the peptide was as efficient at reducing amyloid deposition in Tg2576 animals as either fibrillar Aβ or a multiple Aβ antigen peptide when immunization was initiated at 12 mos. Interestingly, there was significantly less (p < 0.05) activated microglia in the animals immunized with the oligomeric form of Aβ than in those immunized with fAβ. In addition, the antibody response induced by immunization with oligomeric Aβ had a much more restricted epitope response, generating antibodies to oligomeric Aβ, but not to soluble or fibrillar Aβ, similarly to that observed in vaccination of rabbits with the oligomeric conformation of Aβ [23]. The other two immunogens tested, fAβ and MAPAβ_1–33_, induced antibodies to oligomeric, fibrillar and soluble Aβ. Thus, one could speculate that the lower microglia activation is the result of this restricted induction and that this feature may lead to a lower potential for autoimmune responses and inflammatory responses. Further delineation of the basis for the apparently lower microglial activation is, however, necessary to substantiate this hypothesis. Since we did not stain these sections for T cells, it is unknown if the Oligo Aβ immunogen elicited a strong T cell mediated immune response beyond the induction of anti-oligomeric antibody. While this is a concern since a fraction of patients in the human amyloid immunization trial (AN1792) developed meningoencephalitis [7-9,37], any reduction of potentially adverse effects of immunization is desirable.

In contrast, no significant decrease was seen in any pathological marker measured in mice vaccinated with any peptide form when vaccination was initiated at 16 months, a time at which there are high levels of accumulated plaques. The lack of efficacy in reduction of plaque associated pathology by immunization of these older mice, similar to that reported by Das, et al [3], was not due to lower anti-Aβ titers of antibodies in the mice as the levels of reactivity were either equivalent or only slightly lower than those of the mice immunized at an earlier age (12 mo) suggesting age related alterations/differences in trafficking of amyloid out of the brain (see below) or antibodies into the CNS via BBB (or to antibody-independent clearance mechanisms). While it has been hypothesized that older plaques are refractory to degradation and clearance, intracranial injections of anti Aβ antibodies in old APP/Tg mice (16–20 month-old) have been reported to clear long established plaques [38]. Interestingly, Dodart and colleagues reported that passive peripheral immunization of 24 month old PDAPP mice had no effect on amyloid deposition in contrast to similar immunization of younger mice. However, in these very old PDAPP mice significant improvement of behavioral tasks were demonstrated [39]. While the results here clearly demonstrate the lack of effect on clearance of amyloid deposits in mice immunized at an older age, it remains to be seen if improvement in learning can be detected in the very old Tg2576 mice actively immunized with specific conformations of Aβ (particularly the oligomeric conformation), and whether effects of human Aβ immunization are similarly influenced by age and/or plaque deposition at the time of immunization.

In this study we used a second, novel method for quantification of immunohistochemical detection, specifically of amyloid deposits. Although image analysis can bring accuracy and objectivity to IHC, certain drawbacks have historically limited its use. Not only are the processes of image acquisition and data management labor-intensive, but the mechanics of analysis itself can introduce some subjectivity. Automated digital imaging systems, such as the one tested here, have been developed to address such drawbacks. Results from analysis of amyloid burden using such an automated platform correlated tightly with those obtained with an accepted more traditional image analysis approach. Thus, this method is shown to accurately assess amyloid burden in transgenic mouse models of AD. In addition, the use of object-based analysis provides data not obtainable through simple computation of % area positive. For example, immunization with MAPAβ_1–33 _resulted in similar 2.63- and 2.72-fold reductions in Aβ-Positive Object Density (i.e. plaque number) and Aβ-Positive Object Area Ratio (i.e. plaque area), respectively. In contrast, fibrillar Aβ immunization affected these parameters somewhat differently, resulting in a 2.73-fold reduction in Aβ-Positive Object Density but a lower (though still significant) 1.96-fold reduction in Aβ-Positive Object Area Ratio. This raises the intriguing possibility that different immunization paradigms might effect change in amyloid burden preferentially through alteration in either plaque number or in plaque size, although additional study will be required to rigorously test such an assertion.

Interestingly, plaque-associated IgG was detected only in brain of animals immunized with fibrillar Aβ from 12 to 16 months old. While this is consistent with the lack of anti-fibrillar amyloid antibody in the Oligo Aβ immunization scheme, sera of all animals immunized with MAPAβ_1–33 _contained levels of anti-fibrillar Aβ similar to the fAβ immunized animals, but no IgG was detected on amyloid plaques even in the younger set (immunization initiated at 12 m of age) of animals (n = 6) in which reduction of plaques and associated pathology was comparable to fAβ immunized animals. These data suggest that there may be differences in access of the antibody to brain tissue via blood brain barrier or IgG-transport systems in mice immunized with fAβ (perhaps due to antibody epitope specificity [40]). Multiple injections of pertussis toxin have been reported to result in infiltration of immune cells into the brain [29]. However, while PT was given to the MAPAβ_1–33 _mice at the time of initial injection and 24 hours later, these animals showed no immunoglobulin associated with the remaining plaques and similar levels of reduction of plaques in an age specific manner was seen with the Oligo Aβ and fAβ immunogens. However, we did not stain these sections for T cells and therefore cannot confirm nor refute the previously published observation of vascular associated T cells by Furlan and colleagues [29].

Finally, the amount of C3 activation fragments deposited on the plaques in all animals was largely correlated with the amount of thioflavine staining detected in each animal. While this would be expected since fibrillar amyloid has been shown to activate complement [41-43], it is somewhat surprising that there is no apparent increase in those plaques with associated IgG (fAβ immunized, 12–16 mo), since immune complexes (here Aβ/anti Aβ) avidly bind C1q and activate complement. However, additional investigations will be necessary to directly determine the extent of complement activation in each animal treatment group. Indeed increased complement activation is predicted to enhance removal of complement coated plaques (via C3 activation fragments, C3b and iC3b) and thus, whether those plaques remaining are less opsonized or display some other distinguishing characteristic remains to be determined.

The mechanism by which immunization schemes decrease neuropathology and prevent or reverse behavior deficits has not yet been defined. A decrease in amyloid deposition as a result of active immunization was seen in APP overexpressing transgenic animals that are FcR gamma chain deficient [44], suggesting that FcR mediated ingestion by phagocytic cells is not a requirement. However, as stated above, complement activation fragments (particularly C3b and iC3b) are also capable of mediating particle ingestion and thus, complement receptors and/or other phagocytic receptors may provide a mechanism for enhanced ingestion in the absence of FcR. Bacskai et al. demonstrated clearance of plaques by passive immunization of Fab anti amyloid antibody fragments [13] which lack the Fc portion of the antibody molecule and therefore can neither engage Fc receptors nor activate complement, thus providing support for multiple alternative clearance mechanisms. Additional possible mechanisms by which both passive and active immunization may be advantageous have been proposed. The "peripheral sink" model suggests amyloid is cleared in the periphery after being transported from the brain across the vasculature into the blood [12,45]. If therapeutically relevant concentrations of anti Aβ antibodies do not cross the blood brain barrier (BBB), then a mechanism for transporting Aβ out of the brain across the BBB is required [46]. The principle molecule that appears to be involved in transport of Aβ out of the brain across the BBB is the low-density lipoprotein receptor-protein-1 (LRP-1) [47-49], although other molecules such as ApoE and α2- macroglobulin may be involved in this process. Antibodies specific to LRP-1 substantially inhibited the clearance of Aβ_40 _from the brain supporting the role of LRP-1 as a transporter of Aβ peptide out of the brain [47]. Moreover, APP transgenic mice crossed to receptor-associated protein knockout mice (RAP-/-) mice, which are deficient in LRP-1 function, develop increased extracellular Aβ deposition and neurodegeneration [50]. Interestingly, the LRP-1 expression in the BBB appears to decrease significantly with normal aging and in AD. Whether this occurs in aging mice and contributes to the lack of clearance reported here remains to be tested.

Another possible mechanism proposed is that antibodies inhibit fibrillization of amyloid and/or promote depolymerization of fibrils (reviewed in [51]). Still others suggest antibodies must be reactive with the Aβ intermediates to provide their functional protective effect. Wisniewski and colleagues reported that the induction of a predominant IgM anti Aβ response also resulted in behavioral improvements in the Tg2576 animal which did not necessarily correspond to Aβ load in the brain, although high IgM titers did correlate with low amyloid burden [52]. Active immunization of rats with mixed conformations of Aβ peptides prevented the inhibition of LTP activity when conditioned media containing Aβ oligomers were injected intracerebroventricularly, and this prevention of inhibition of LTP correlated with antibody recognition of the Aβ oligomers [24]. Anti-oligomeric antibody induced here could promote therapeutic clearance and removal of amyloid via any of these mechanisms, and by limiting the immune response, may limit detrimental activation and/or promotion of inflammation.

A hypothesis consistent with the observations in both human AD and transgenic mouse models of AD is that there are at least two, likely overlapping, stages contributing to neuronal dysfunction in AD. Early events involving the intracellular accumulation of Aβ peptides [53] and/or generation of oligomeric Aβ peptides [21,23] or complexes containing oligomeric Aβ [54] would lead to neuronal alterations and cellular death. Factors arising during aging such as oxidative stress, mitochondrial dysfunction, deficiencies in lysosomal function or regulation of neurotrophic factors that lead to processing outcomes which are harmful to the cell may contribute to, or enhance, susceptibility to stress induced by Aβ [55-57]. Increased extracellular fibrillar Aβ deposits from these processes and/or overload of the phagocytic capacity of the local region provide a nidus for complement activation [41,42]. The generation of the proinflammatory complement activation products would initiate a secondary phase of inflammatory events that accelerate local neuronal damage, loss, and decline of cognitive function [34]. Neuronal injury at both stages could be avoided or diminished by a decrease in amyloid peptide in the brain, a direct goal of immunotherapy. Providing increased specificity for the immune response however, may also decrease the probability of activating detrimental inflammatory responses, and thus is a potential advantage of the oligomeric amyloid conformation as the immunogen.

## Conclusion

In summary, immunization with any amyloid peptide immunogen resulted in significant reduction (45–55%) of plaque pathology and decreased inflammatory microglial cell reactivity (40–65%) only when immunized prior to the massive deposition of large mature plaques. These decreases in pathology did not necessarily correlate with the level of anti Aβ1–42 IgG in sera or with plaque-associated IgG. This first analysis of deposition/accumulation of complement C3 activation within an immunization trial shows association of C3b/iC3b with plaques that correlates with the amount of thioflavine plaques present rather than any association with the antibody response induced. Finally, immunization with oligomeric Aβ1–42 induced a greater decrease in "reactive" microglia relative to immunization with fibrillar Aβ as determined by microglial surface expression of CD45 and MAC-1 antigens. The lower microglial response resulting from immunization with oligomeric Aβ suggests that the goal of depleting plaques can be accomplished while inducing less neuroinflammation, and thus facilitating the application of immunotherapy to treatment and/or prevention of AD in humans.

## Abbreviations

AD, Alzheimer's Disease; BBB, blood brain barrier; CFA, complete Freund's adjuvant; fAβ, fibrillar amyloid peptide 1–42; IFA, incomplete Freund's adjuvant; oligo Aβ, Aβ 1–42 conjugated to colloidal gold; PT, pertussis toxin.

## Competing interests

Charles Glabe and Rakez Kayed are consultants for Kinexis, Inc. and Scott Webster is an employee at Clarient, Inc. The authors declare that they have no other competing interests.

## Authors' contributions

JZ performed all animal treatments, perfusions, immunohistochemistry, image analysis, data analysis and presentation, and guided or performed tissue preparation and serum collection. MF performed immunohistochemistry, image analysis, data analysis and presentation, molecular genetic studies and manuscript preparation; RK prepared immunogens and performed immunoassays, S.W. performed image analysis, data analysis and manuscript preparation; IH performed immunohistochemistry, image analysis, data analysis and presentation and assisted with perfusions and animal care; OY performed immunohistochemistry and image analysis; DC assisted with the design of the study and manuscript preparation; CG contributed to the design of the study and data analysis, and manuscript preparation. AJT developed the design of the study, guided study execution, directed data analysis, interpretation and presentation, and drafted and edited the manuscript. All authors read and approved the final manuscript.

## References

[B1] Hardy J, Selkoe DJ (2002). The amyloid hypothesis of Alzheimer's disease: progress and problems on the road to therapeutics. Science.

[B2] Golde TE (2005). The Abeta hypothesis: leading us to rationally-designed therapeutic strategies for the treatment or prevention of Alzheimer disease. Brain Pathol.

[B3] Das P, Murphy MP, Younkin LH, Younkin SG, Golde TE (2001). Reduced effectiveness of Abeta1-42 immunization in APP transgenic mice with significant amyloid deposition. Neurobiol Aging.

[B4] Schenk D, Barbour R, Dunn W, Gordon G, Grajeda H, Guido T, Hu K, Huang JP, Johnson-Wood K, Khan K, Kholodenko D, Lee M, Liao ZM, Lieberburg I, Motter R, Mutter L, Soriano F, Shopp G, Vasquez N, Vandevert C, Walker S, Wogulis M, Yednock T, Games D (1999). Immunization with amyloid-b attenuates Alzheimer disease-like pathology in the PDAPP mouse. Nature.

[B5] Morgan D, Diamond DM, Gottschall PE, Ugen KE, Dickey C, Hardy J, Duff K, Jantzen P, DiCarlo G, Wilcock D, Connor K, Hatcher J, Hope C, Gordon M, Arendash GW (2000). A beta peptide vaccination prevents memory loss in an animal model of Alzheimer's disease. Nature.

[B6] Janus C, Pearson J, McLaurin J, Mathews PM, Jiang Y, Schmidt SD, Chishti MA, Horne P, Heslin D, French J, Mount HT, Nixon RA, Mercken M, Bergeron C, Fraser PE, George-Hyslop P, Westaway D (2000). A beta peptide immunization reduces behavioural impairment and plaques in a model of Alzheimer's disease. Nature.

[B7] Nicoll JA, Wilkinson D, Holmes C, Steart P, Markham H, Weller RO (2003). Neuropathology of human Alzheimer disease after immunization with amyloid-beta peptide: a case report. Nat Med.

[B8] Orgogozo JM, Gilman S, Dartigues JF, Laurent B, Puel M, Kirby LC, Jouanny P, Dubois B, Eisner L, Flitman S, Michel BF, Boada M, Frank A, Hock C (2003). Subacute meningoencephalitis in a subset of patients with AD after Abeta42 immunization. Neurol.

[B9] Ferrer I, Boada RM, Sanchez Guerra ML, Rey MJ, Costa-Jussa F (2004). Neuropathology and pathogenesis of encephalitis following amyloid-beta immunization in Alzheimer's disease. Brain Pathol.

[B10] McGeer PL, McGeer E (2003). Is there a future for vaccination as a treatment for Alzheimer's disease?. Neurobiol Aging.

[B11] Oddo S, Billings L, Kesslak JP, Cribbs DH, LaFerla FM (2004). Abeta immunotherapy leads to clearance of early, but not late, hyperphosphorylated tau aggregates via the proteasome. Neuron.

[B12] DeMattos RB, Bales KR, Cummins DJ, Dodart JC, Paul SM, Holtzman DM (2001). Peripheral anti-A beta antibody alters CNS and plasma A beta clearance and decreases brain A beta burden in a mouse model of Alzheimer's disease. Proc Natl Acad Sci U S A.

[B13] Bacskai BJ, Kajdasz ST, McLellan ME, Games D, Seubert P, Schenk D, Hyman BT (2002). Non-Fc-mediated mechanisms are involved in clearance of amyloid-beta in vivo by immunotherapy. J Neurosci.

[B14] Bard F, Cannon C, Barbour R, Burke RL, Games D, Grajeda H, Guido T, Hu K, Huang J, Johnson-Wood K, Khan K, Kholodenko D, Lee M, Lieberburg I, Motter R, Nguyen M, Soriano F, Vasquez N, Weiss K, Welch B, Seubert P, Schenk D, Yednock T (2000). Peripherally administered antibodies against amyloid beta-peptide enter the central nervous system and reduce pathology in a mouse model of Alzheimer disease. Nat Med.

[B15] Wilcock DM, DiCarlo G, Henderson D, Jackson J, Clarke K, Ugen KE, Gordon MN, Morgan D (2003). Intracranially administered anti-abeta antibodies reduce Beta -amyloid deposition by mechanisms both independent of and associated with microglial activation. J Neurosci.

[B16] Wilcock DM, Rojiani A, Rosenthal A, Subbarao S, Freeman MJ, Gordon MN, Morgan D (2004). Passive immunotherapy against Abeta in aged APP-transgenic mice reverses cognitive deficits and depletes parenchymal amyloid deposits in spite of increased vascular amyloid and microhemorrhage. J Neuroinflammation.

[B17] Billings LM, Oddo S, Green KN, McGaugh JL, LaFerla FM (2005). Intraneuronal Abeta causes the onset of early Alzheimer's disease-related cognitive deficits in transgenic mice. Neuron.

[B18] Lemere CA, Spooner ET, Leverone JF, Mori C, Iglesias M, Bloom JK, Seabrook TJ (2003). Amyloid-beta immunization in Alzheimer's disease transgenic mouse models and wildtype mice. Neurochem Res.

[B19] Bard F, Barbour R, Cannon C, Carretto R, Fox M, Games D, Guido T, Hoenow K, Hu K, Johnson-Wood K, Khan K, Kholodenko D, Lee C, Lee M, Motter R, Nguyen M, Reed A, Schenk D, Tang P, Vasquez N, Seubert P, Yednock T (2003). Epitope and isotype specificities of antibodies to beta -amyloid peptide for protection against Alzheimer's disease-like neuropathology. Proc Natl Acad Sci U S A.

[B20] Austin L, Arendash GW, Gordon MN, Diamond DM, DiCarlo G, Dickey C, Ugen K, Morgan D (2003). Short-term beta-amyloid vaccinations do not improve cognitive performance in cognitively impaired APP + PS1 mice. Behav Neurosci.

[B21] Walsh DM, Klyubin I, Fadeeva JV, Cullen WK, Anwyl R, Wolfe MS, Rowan MJ, Selkoe DJ (2002). Naturally secreted oligomers of amyloid beta protein potently inhibit hippocampal long-term potentiation in vivo. Nature.

[B22] Wang HW, Pasternak JF, Kuo H, Ristic H, Lambert MP, Chromy B, Viola KL, Klein WL, Stine WB, Krafft GA, Trommer BL (2002). Soluble oligomers of beta amyloid (1-42) inhibit long-term potentiation but not long-term depression in rat dentate gyrus. Brain Res.

[B23] Kayed R, Head E, Thompson JL, McIntire TM, Milton SC, Cotman CW, Glabe CG (2003). Common structure of soluble amyloid oligomers implies common mechanism of pathogenesis. Science.

[B24] Klyubin I, Walsh DM, Lemere CA, Cullen WK, Shankar GM, Betts V, Spooner ET, Jiang L, Anwyl R, Selkoe DJ, Rowan MJ (2005). Amyloid beta protein immunotherapy neutralizes Abeta oligomers that disrupt synaptic plasticity in vivo. Nat Med.

[B25] Kotilinek LA, Bacskai B, Westerman M, Kawarabayashi T, Younkin L, Hyman BT, Younkin S, Ashe KH (2002). Reversible memory loss in a mouse transgenic model of Alzheimer's disease. J Neurosci.

[B26] Selkoe DJ, Schenk D (2003). Alzheimer's disease: molecular understanding predicts amyloid-based therapeutics. Annu Rev Pharmacol Toxicol.

[B27] Klein WL (2002). Abeta toxicity in Alzheimer's disease: globular oligomers (ADDLs) as new vaccine and drug targets. Neurochem Int.

[B28] Hsiao KK, Chapman P, Nilsen S, Eckman C, Harigaya Y, Younkin S, Yang F, Cole G (1996). Correlative memory deficits, Ab elevations, and amyloid plaques in transgenic mice.. Science.

[B29] Furlan R, Brambilla E, Sanvito F, Roccatagliata L, Olivieri S, Bergami A, Pluchino S, Uccelli A, Comi G, Martino G (2003). Vaccination with amyloid-beta peptide induces autoimmune encephalomyelitis in C57/BL6 mice. Brain.

[B30] Cummings JL, Vinters HV, Cole GM, Khachaturian ZS (1998). Alzheimer's disease: etiologies, pathophysiology, cognitive reserve, and treatment opportunities. Neurol.

[B31] LeVine HIII (1999). Quantification of beta-sheet amyloid fibril structures with thioflavin T. Methods Enzymol.

[B32] Aloisi F (2001). Immune function of microglia. Glia.

[B33] Streit WJ, Mrak RE, Griffin WS (2004). Microglia and neuroinflammation: a pathological perspective. J Neuroinflammation.

[B34] Fonseca MI, Zhou J, Botto M, Tenner AJ (2004). Absence of C1q leads to less neuropathology in transgenic mouse models of Alzheimer's disease. J Neurosci.

[B35] Matsuoka Y, Picciano M, Malester B, LaFrancois J, Zehr C, Daeschner JM, Olschowka JA, Fonseca MI, O'Banion MK, Tenner AJ, Lemere CA, Duff K (2001). Inflammatory responses to amyloidosis in a transgenic mouse model of Alzheimer's disease. Am J Pathol.

[B36] Mastellos D, Prechl J, Laszlo G, Papp K, Olah E, Argyropoulos E, Franchini S, Tudoran R, Markiewski M, Lambris JD, Erdei A (2004). Novel monoclonal antibodies against mouse C3 interfering with complement activation: description of fine specificity and applications to various immunoassays. Mol Immunol.

[B37] Bayer AJ, Bullock R, Jones RW, Wilkinson D, Paterson KR, Jenkins L, Millais SB, Donoghue S (2005). Evaluation of the safety and immunogenicity of synthetic Abeta42 (AN1792) in patients with AD. Neurol.

[B38] Wilcock DM, Rojiani A, Rosenthal A, Levkowitz G, Subbarao S, Alamed J, Wilson D, Wilson N, Freeman MJ, Gordon MN, Morgan D (2004). Passive amyloid immunotherapy clears amyloid and transiently activates microglia in a transgenic mouse model of amyloid deposition. J Neurosci.

[B39] Dodart JC, Bales KR, Gannon KS, Greene SJ, DeMattos RB, Mathis C, DeLong CA, Wu S, Wu X, Holtzman DM, Paul SM (2002). Immunization reverses memory deficits without reducing brain Abeta burden in Alzheimer's disease model. Nat Neurosci.

[B40] Racke MM, Boone LI, Hepburn DL, Parsadainian M, Bryan MT, Ness DK, Piroozi KS, Jordan WH, Brown DD, Hoffman WP, Holtzman DM, Bales KR, Gitter BD, May PC, Paul SM, DeMattos RB (2005). Exacerbation of cerebral amyloid angiopathy-associated microhemorrhage in amyloid precursor protein transgenic mice by immunotherapy is dependent on antibody recognition of deposited forms of amyloid beta. J Neurosci.

[B41] Rogers J, Cooper NR, Webster S, Schultz J, McGeer PL, Styren SD, Civin WH, Brachova L, Bradt B, Ward P, Lieberburg I (1992). Complement activation by beta-amyloid in Alzheimer disease.. Proc Natl Acad Sci.

[B42] Jiang H, Burdick D, Glabe CG, Cotman CW, Tenner AJ (1994). b-amyloid activates complement by binding to a specific region of the collagen-like domain of the C1q A chain. J Immunol.

[B43] Bradt BM, Kolb WP, Cooper NR (1998). Complement-dependent proinflammatory properties of the Alzheimer's disease b-peptide.. J Exp Med.

[B44] Das P, Howard V, Loosbrock N, Dickson D, Murphy MP, Golde TE (2003). Amyloid-beta immunization effectively reduces amyloid deposition in FcRgamma-/- knock-out mice. J Neurosci.

[B45] Lemere CA, Spooner ET, LaFrancois J, Malester B, Mori C, Leverone JF, Matsuoka Y, Taylor JW, DeMattos RB, Holtzman DM, Clements JD, Selkoe DJ, Duff KE (2003). Evidence for peripheral clearance of cerebral Abeta protein following chronic, active Abeta immunization in PSAPP mice. Neurobiol Dis.

[B46] Holtzman DM, Bales KR, Paul SM, DeMattos RB (2002). Abeta immunization and anti-Abeta antibodies: potential therapies for the prevention and treatment of Alzheimer's disease. Adv Drug Deliv Rev.

[B47] Shibata M, Yamada S, Kumar SR, Calero M, Bading J, Frangione B, Holtzman DM, Miller CA, Strickland DK, Ghiso J, Zlokovic BV (2000). Clearance of Alzheimer's amyloid-ss(1-40) peptide from brain by LDL receptor-related protein-1 at the blood-brain barrier. J Clin Invest.

[B48] Deane R, Wu Z, Sagare A, Davis J, Du YS, Hamm K, Xu F, Parisi M, LaRue B, Hu HW, Spijkers P, Guo H, Song X, Lenting PJ, Van Nostrand WE, Zlokovic BV (2004). LRP/amyloid beta-peptide interaction mediates differential brain efflux of Abeta isoforms. Neuron.

[B49] Zlokovic BV (2004). Clearing amyloid through the blood-brain barrier. J Neurochem.

[B50] Van Uden E, Mallory M, Veinbergs I, Alford M, Rockenstein E, Masliah E (2002). Increased extracellular amyloid deposition and neurodegeneration in human amyloid precursor protein transgenic mice deficient in receptor-associated protein. J Neurosci.

[B51] Schenk D, Hagen M, Seubert P (2004). Current progress in beta-amyloid immunotherapy. Curr Opin Immunol.

[B52] Sigurdsson EM, Knudsen E, Asuni A, Fitzer-Attas C, Sage D, Quartermain D, Goni F, Frangione B, Wisniewski T (2004). An attenuated immune response is sufficient to enhance cognition in an Alzheimer's disease mouse model immunized with amyloid-beta derivatives. J Neurosci.

[B53] Echeverria V, Cuello AC (2002). Intracellular A-beta amyloid, a sign for worse things to come?. Mol Neurobiol.

[B54] Lambert MP, Barlow AK, Chromy BA, Edwards C, Freed R, Liosatos M, Morgan TE, Rozovsky I, Trommer B, Viola KL, Wals P, Zhang C, Finch CE, Krafft GA, Klein WL (1998). Diffusible, nonfibrillar ligands derived from Abeta1-42 are potent central nervous system neurotoxins. Proc Natl Acad Sci U S A.

[B55] Nixon RA, Cataldo AM, Mathews PM (2000). The endosomal-lysosomal system of neurons in Alzheimer's disease pathogenesis: a review. Neurochem Res.

[B56] Kujoth GC, Hiona A, Pugh TD, Someya S, Panzer K, Wohlgemuth SE, Hofer T, Seo AY, Sullivan R, Jobling WA, Morrow JD, Van RH, Sedivy JM, Yamasoba T, Tanokura M, Weindruch R, Leeuwenburgh C, Prolla TA (2005). Mitochondrial DNA mutations, oxidative stress, and apoptosis in mammalian aging. Science.

[B57] Mattson MP, Maudsley S, Martin B (2004). BDNF and 5-HT: a dynamic duo in age-related neuronal plasticity and neurodegenerative disorders. Trends Neurosci.

[B58] Viale G, Gambacorta M, Coggi G, Dell'Orto P, Milani M, Doglioni C (1991). Glial fibrillary acidic protein immunoreactivity in normal and diseased human breast. Virchows Arch A Pathol Anat Histopathol.

[B59] Jung SS, Gauthier S, Cashman NR (1999). Beta-amyloid precursor protein is detectable on monocytes and is increased in Alzheimer's disease. Neurobiol Aging.

[B60] Anderson DC, Miller LJ, Schmalstieg FC, Rothlein R, Springer TA (1986). Contributions of the Mac-1 glycoprotein family to adherence-dependent granulocyte functions:  Structure-function assessments employing subunit-specific monoclonal antibodies.. J Immunol.

[B61] Brewer Y, Palmer A, Taube D, Welsh K, Bewick M, Bindon C, Hale G, Waldmann H, Dische F, Parsons V, . (1989). Effect of graft perfusion with two CD45 monoclonal antibodies on incidence of kidney allograft rejection. Lancet.

[B62] Yang AJ, Knauer M, Burdick DA, Glabe C (1995). Intracellular A beta 1-42 aggregates stimulate the accumulation of stable, insoluble amyloidogenic fragments of the amyloid precursor protein in transfected cells. J Biol Chem.

